# Methodological quality and recommendations of hemophilia clinical practice guidelines: A scoping review

**DOI:** 10.1002/hsr2.1326

**Published:** 2023-07-13

**Authors:** Carolina J. Delgado‐Flores, David García‐Gomero, Liseth Pinedo‐Castillo, Alvaro Taype‐Rondan

**Affiliations:** ^1^ Carrera de Farmacia y Bioquímica, Facultad de Ciencias de la Salud Universidad Científica del Sur Lima Perú; ^2^ Facultad de Medicina “San Fernando” Universidad Nacional Mayor de San Marcos Lima Peru; ^3^ Escuela Profesional de Medicina Humana de la Universidad Señor de Sipán Chiclayo Peru; ^4^ Asociación Científica de Estudiantes de Medicina de la Universidad Señor de Sipán Chiclayo Peru; ^5^ Universidad San Ignacio de Loyola Unidad de Investigación para la Generación y Síntesis de Evidencias en Salud Lima Peru; ^6^ EviSalud – Evidencias en Salud Lima Peru

**Keywords:** clinical practice guidelines, clinical guidelines, hemophilia, prophylaxis dose

## Abstract

**Background and Aims:**

Hemophilia clinical practice guidelines (CPGs) play a vital role in guiding healthcare professionals' decisions. However, the quality and recommendations of CPGs for hemophilia may vary. This study aimed to assess the methodological quality of hemophilia CPGs published between 2017 and 2021 and compare their recommendations for prophylaxis using clotting factor concentrate.

**Methods:**

We conducted a comprehensive search for relevant CPGs in PubMed, TripDatabase, Grades of Recommendation, Assessment, Development, and Evaluation (GRADE) International Guidelines Database, Google Scholar, and Google. We used the AGREE‐II instrument to assess the methodological quality of each CPG and compared their recommendations for prophylaxis.

**Results:**

Of the 11 CPGs that met the inclusion criteria, 5/11 were developed in upper‐middle‐income countries, and 6/11 used the GRADE methodology. The primary prophylaxis dose recommendations varied among the CPGs, with 4/11 recommending a low dose, 6/11 recommending an intermediate or high dose, and 1/11 not issuing a recommendation. However, only 2/11 CPGs provided justification for their recommendations on initiation and dose, and no economic evaluations were conducted to support these recommendations.

**Conclusion:**

The quality of hemophilia CPGs is not optimal, with inconsistent recommendations for prophylaxis and a lack of justification for these recommendations. To ensure evidence‐based and trustworthy recommendations, there is a need for transparency and improvement in the decision‐making process of hemophilia CPGs.

## Key points

1. This study assessed the methodological quality of hemophilia clinical practice guidelines (CPGs) and compare their recommendations for prophylaxis using clotting factor concentrate.
2. Few hemophilia CPGs (3/11) met the criteria for adequate methodological quality as assessed by the AGREE II instrument.
3. 2/11 CPGs provided justifications for their prophylaxis dose recommendations, and none of them conducted economic evaluations to support these recommendations.

## 1 INTRODUCTION

Hemophilia is an inherited bleeding disorder caused by a deficiency of coagulation factor VIII (hemophilia A) or IX (hemophilia B), both X‐linked recessive disorders.^1^ Clinical manifestations relate to bleeding, bleeding sequelae, or complications of coagulation factor infusion.^2^ It affects more than 1.2 million individuals worldwide, almost exclusively males.^3^ Hemophilia A occurs in approximately 1 in 4000 to 5000 live male births, while Hemophilia B occurs in around 1 in 15,000 to 1 in 30,000 live male births. Severe disease is more common in hemophilia A.^4^

The management of hemophilia is based on deficient clotting factor concentrates or by‐pass agents when inhibitors are present. Indeed, factor concentrate is indicated as a prophylaxis measure to prevent long‐term complications such as hemophilic arthropathy.^5^

Clinical practice guidelines (CPGs) issue recommendations regarding which patients should receive primary prophylaxis with clotting factor concentrates and in what dose. However, these recommendations could be heterogeneous across countries due to differences in contexts and available resources and different methodologies used to reach evidence‐based recommendations.

Thus, this study aims to perform a scoping review of the CPGs that address the management of hemophilia, assess their methodological quality, and compare their recommendations issued for prophylaxis with clotting factor concentrate.

## 2 MATERIAL AND METHODS

We conducted a scoping review of CPGs for hemophilia, following the preferred reporting items for systematic reviews and meta‐analyses guidelines for scoping reviews (PRISMA‐ScR) to ensure the adequate reporting of this study.^6^

### 2.1 Eligibility criteria

We included CPGs that provided recommendations for the management of hemophilia, published between 2017 and 2021, and available in full text. We did not apply any language restrictions.

### 2.2 Search strategy

During December 2021, we searched in five sources: Pubmed, TripDataBase, Grades of Recommendation, Assessment, Development, and Evaluation (GRADE) International Guidelines Database, Google Scholar, and Google. The search strategies included terms such as “hemophilia,” “guideline,” and “recommendation” (Supporting Information: Table S1).

### 2.3 CPGs selection and data extraction

Two researchers independently reviewed the documents to assess their eligibility (C. D. F. and D. G. G.). Discrepancies were resolved with a third researcher (A. T. R.).

Two authors performed data extraction independently (C. D. F. and L. P. C. for general data, and C. D. F. and D. G. G. for recommendations data) in Microsoft Excel spreadsheets. They extracted the following data for each CPG: general data (year, institution, country, income level of the country according to World Bank Data,^7^ funding, involvement of patients or their representatives, methodology used to reach recommendations, evidence review, Summary of Findings [SoF] or Evidence Profile [EP] tables, and characteristics of the prophylaxis dose recommendation) and recommendations data (criteria for starting primary prophylaxis, dose category, criteria for dose selection, and criteria for prophylaxis discontinuation).

We standardized the prophylaxis doses according to the World Federation of Hemophilia classification: low‐dose (20 to <45 IU/kg per week), intermediate‐dose (45 to <75 IU/kg per week), high‐dose ( ≥ 75  IU/kg per week).^5^

### 2.4 Methodological quality assessment

We used the Appraisal of Guidelines for Research & Evaluation Instrument–II (AGREE‐II) instrument to assess the methodological quality of the included CPGs. This instrument has 23 items in six domains (scope and purpose, stakeholder involvement, rigor of development, clarity and presentation, applicability, and editorial independence). Each CPG was rated by two authors (C. D. F. and L. P. C.). When the scores for a certain item had a difference greater than two points, we discussed the criteria of the item to reach a consensus. Then, we used the mean score for each item and followed the AGREE‐II instrument guideline to calculate the score for each domain.^8^

We used a cut‐off of ≥ 60% to define an acceptable quality for each domain of the AGREE‐II Instrument and the total score. We took this cut‐off point from previous studies.^9^, ^10^, ^11^, ^12^, ^13^, ^14^

### 2.5 Analyses

We used absolute and relative frequencies to present the results. Also, we performed literal transcriptions or summarized the recommendations of interest.

### 2.6 Ethics statement

This study does not involve human participants or animal subjects, and therefore, does not require institutional review board (IRB) approval.

## 3 RESULTS

**Figure 1:**
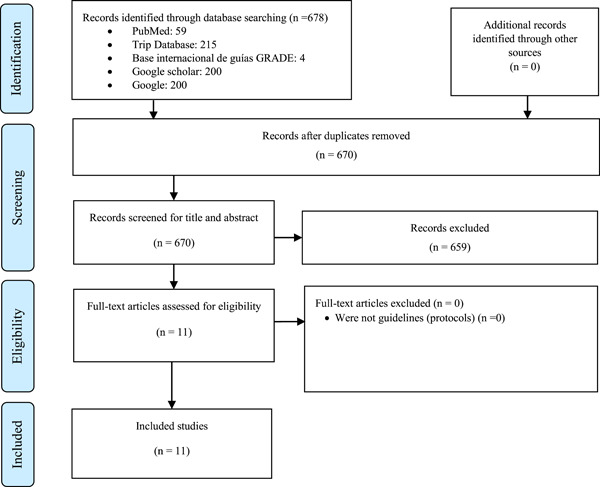
Flow diagram (study selection).

We found 678 records in database searching. After duplicates removal, we screened 670 records and finally included 11 hemophilia CPG that issued prophylaxis recommendations, published or updated between 2017 and 2021 (Figure 1).

**Table 1:** Characteristics of the included clinical practice guidelines for hemophilia.

No	Author year (country)	Scope	Funding/Multidiscipli‐nary team	Involvement of patients or their representatives	Methodology used to reach recommendations	Conducting evidence reviews	Showed tables SoF or EP	For the “primary prophylaxis dose” recommendation			
								Mentions search strategy	List of included studies	Justification of the dose decision (benefits and harms caused)	Did an economic evaluation comparing different doses
*CPGs from lower‐middle‐income countries*											
1	IAP 2018 (India)	Diagnosis and management in pediatric patients with hemophilia	Not reported/Not clear	No	Not clear	No	No	No	No	No	No
2	GMHE 2018 (Egypt)	Management of people with hemophilia	Pfizer/No	No	Not clear	No	No	No	No	No	No
*CPGs from upper‐middle‐income countries*											
3	GMHC 2021 (Argentina)	Treatment in patients without inhibitors and management of patients with inhibitors	Not reported/No	No	Not clear	No	No	No	No	No	No
4	MINSAL 2020 (Chile)	Diagnosis and management of hemophilia	Ministerio de Salud de Chile/Si	Yes	GRADE methodology	Yes	Yes	Yes	Yes	No	No
5	IMSS 2018 (Mexico)	Diagnosis and management of hereditary hemophilia in <16	Instituto Mexicano del Seguro Social/No	No	GRADE methodology	Yes	No	Yes	Yes	No	No
6	IGSS 2017 (Guatemala)	Diagnosis and management in pediatric patients with hemophilia	Instituto Guatemalteco de Seguridad Social/No	No	GRADE methodology	Yes	No	No	Yes	No	No
7	EsSalud 2021 (Peru)	Diagnosis and management in patients with hemophilia	Seguridad Social del Perú/Yes	Yes	GRADE methodology	Yes	Yes	Yes	Yes	Yes	No
*CPGs from high‐income countries*											
8	NHG 2021 (Nordic countries)	Diagnosis and management in patients with hemophilia	Not reported/Not clear	No	Not clear	No	No	No	Yes	No	No
9	NHC 2019 (Ireland)	Management of hemophilia patients with inhibitors	Not reported/Yes	No	Not clear	No	No	a	a	a	a
10	BSH 2020 (United Kingdom)	Management of hemophilia patients	Not reported/Not clear	No	GRADE methodology	Yes	No	Yes	Yes	No	No
*International CPGs*											
11	World Federation of Hemophilia (WFH) 2020 (Canada)	Diagnosis and management of hemophilia, in patients with inhibitors and without inhibitors	World Federation of Hemophilia/Yes	Yes	GRADE methodology	Yes	No	No	Yes	Yes	No

Abbreviations: CPG, clinical practice guideline; EP, Evidence Profile; GRADE, Grades of Recommendation, Assessment, Development, and Evaluation; SoF, Summary of Findings.

^a^ No recommendation issued.

From the included guidelines, 3/11^5^, ^15^, ^16^ involved patients or their representatives in the process of development of the CPG, 6/11^5^, ^15^, ^16^, ^17^, ^18^, ^19^ declared to have used the Grades of Recommendation, Assessment, Development, and Evaluation (GRADE) methodology to issue their recommendations, and 2/11^15^, ^16^ reported tables Summary of Findings (SoF). Regarding how recommendations about prophylaxis dose were reached, 4/11^15^, ^16^, ^19^, ^17,19^ mentioned a search strategy, and 2/11^5^, ^15^ showed a justification for the recommendation regarding dose decision (Table 1).

We used the AGREE II instrument to assess the quality of guidelines. The overall assessment score ranged from 14% to 83% (mean: 45%), and 3/11 guidelines had an overall assessment score ≥ 60%.^15^, ^16^, ^17^ Regarding the third domain, scores ranged from 1% to 84% (mean: 35%), and 3/11 guidelines had a score ≥ 60%^15^, ^16^, ^17^ (Table 2).

**Table 2:** Quality appraisal of clinical practice guidelines for hemophilia, using the AGREE‐II instrument.

Author year (country)	Domain 1	Domain 2	Domain 3	Domain 4	Domain 5	Domain 6	Overall assessment
*CPGs from lower‐middle‐income countries*							
IAP 2018 (India)	44%	25%	6%	28%	17%	58%	33%
GMHE 2018 (Egypt)	42%	33%	10%	69%	8%	33%	33%
*CPGs from upper‐middle‐income countries*							
GMHC 2021 (Argentina)	28%	28%	1%	19%	8%	0%	14%
MINSAL 2020 (Chile)	100%	78%	**84%**	94%	58%	92%	**83%**
IMSS 2018 (Mexico)	86%	67%	**67%**	92%	8%	83%	**63%**
EsSalud 2021 (Peru)	94%	92%	**84%**	94%	67%	50%	**80%**
IGSS 2017 (Guatemala)	81%	50%	15%	39%	8%	50%	40%
*CPGs from high‐income countries*							
NHG 2021 (Nordic countries)	47%	17%	14%	67%	8%	0%	24%
NHC 2019 (Ireland)	47%	56%	4%	11%	8%	8%	21%
BSH 2020 (United Kingdom)	86%	33%	41%	58%	8%	67%	53%
*International CPGs*							
World Federation of Hemophilia (WFH) 2020 (International)	47%	53%	56%	67%	13%	67%	48%

*Note*: Bold: ≥ 60%.

### 3.1 Criteria for starting primary prophylaxis

The recommendations regarding prophylaxis start were heterogeneous: while 3/11 CPGs^18^, ^19^, ^20^ recommended starting in moderate or severe hemophilia, 4/11^5^, ^15^, ^17^, ^21^ recommended its initiation in severe hemophilia, 3/11^16^, ^21^, ^22^ did not mention whether severity is a criterion for starting prophylaxis, and 1/11^23^ did not issue a recommendation on this regard. Moreover, other criteria (such as hemorrhagic phenotype, age, hemarthrosis/bleeding, or availability) were heterogeneous across CPGs (Table 3).

**Table 3:** Recommendations regarding prophylaxis with clotting factors in patients with Hemophilia A or B without inhibitors, issued by clinical practice guidelines for hemophilia.

No	Author year (country)	Criteria for starting primary prophylaxis	Dose category	Criteria for dose selection	Prophylaxis continuity
*Lower‐middle income countries*					
1	IAP 2018 (India)	In children, before the second bleeding episode (No mention of the severity)	Low, intermediate, or high dose	Clinical criteria and availability	Does not mention
2	GMHE 2018 (Egypt)	In very young children (No mention of the severity)	Low dose	Clinical criteria	Lifetime prophylaxis
*Upper‐middle income countries*					
3	GMHC 2021 (Argentina)	Severe hemophilia and: − From the age of 2 years if a hemarthrosis has not yet occurred − First/second hemarthrosis, in the absence of osseous‐cartilaginous joint disease − After a central nervous system hemorrhage (in the absence of the two previous situations)	Intermediate or high dose	Clinical criteria and availability	Lifetime prophylaxis
4	MINSAL 2020 (Chile)	In hemorrhagic phenotype: Before 2 years old or at the latest after the first hemarthrosis. (No mention of the severity)	Intermediate or high dose	Does not specify	Does not mention
5	IMSS 2018 (Mexico)	In severe hemophilia, and: − After the first joint hemorrhagic event (elbows, knees, ankles, hips, shoulders) or in the presence of a considerable muscular hematoma. − Before 3 years of age. − Before clinical joint damage. − Following a central nervous system hemorrhage meeting previous criteria.	Intermediate or high dose	Clinical criteria	Does not mention
6	IGSS 2017 (Guatemala)	In moderate or severe hemophilia, with plasma factor activity <2%, before 30 months of age, and without the presence of joint damage	Intermediate or high dose	Clinical criteria	Does not mention
7	EsSalud 2021 (Peru)	In severe hemophilia: at the doctor's discretion or according to the availability of the factor.	Low, intermediate, or high dose	Clinical criteria and availability	Does not mention
*High‐income countries*					
8	NHG 2021 (Nordic countries)	Prophylaxis should be offered to patients with moderate or severe hemophilia with factor levels of 1 to 2 IU/dL; before the first year of life and before the first joint bleed.	Intermediate or high dose	Clinical criteria	Does not mention
9	NHC 2019 (Ireland)	Does not specify	Does not mention	Does not mention	Does not mention
10	BSH 2020 (United Kingdom)	In moderate or severe hemophilia: In young children, as soon as possible or in the presence of hemarthrosis.	Intermediate or high dose	Clinical criteria	Lifetime prophylaxis. In case of discontinuation, resume if there is a deterioration of the quality of life due to frequent bleeding.
*International CPGs*					
11	World Federation of Hemophilia (WFH) 2020 (Canada)	In pediatric patients with severe hemophilia, considering the availability of the factor.	Low, intermediate, or high dose	Clinical criteria and availability	Lifetime prophylaxis. In case of discontinuation, close monitoring.

### 3.2 Dose and criteria for dose selection

Considering the World Federation of Hemophilia (WFH) international guideline classification of doses,^5^ we found that 4/11 CPGs^5^, ^15^, ^22^, ^24^ considered the possibility of starting prophylaxis from a low dose, 6/11 CPGs^16^, ^17^, ^18^, ^19^, ^20^, ^21^ considered starting prophylaxis from an intermediate or high dose, and 1/11 CPG^23^ did not issue a recommendation on this regard (Table 3).

Regarding dose individualization, 7/11 guidelines recommended dose selection or progressive increase based on clinical criteria (such as disease severity or bleeding behavior), 2/11^15^, ^21^ considered factor availability and clinical characteristics as a criterion for dose selection, and 2/11^16^, ^23^ do not specify any criteria for dose selection (Table 3).

### 3.3 Criteria for discontinuing prophylaxis

Regarding prophylaxis discontinuation, three CPGs^19^, ^21^, ^24^ considered lifelong prophylaxis as a standard of care, 1/11^5^ recommended lifelong prophylaxis but stated that continued monitoring should be performed in case of discontinuation, and the other 7/11 CPGs did not issue a recommendation regarding this topic (Table 3).

### 3.4 On‐demand treatment

Regarding bleeding without immediate life‐threatening, 5/11 CPGs^5^, ^15^, ^16^, ^17^, ^22^ recommended raising the plasma concentration of the factor to 40–60 IU/dL (2–5 days or more, according to the response), 1/11^21^ recommended raising the plasma concentration of the factor to ≥ 30 IU/dL, for 2–3 days, 1/11^23^ recommended raising the plasma concentration of the factor to 50  IU/dL (without specifying time), and 4/11^18^, ^19^, ^20^, ^24^ did not issue a recommendation on this regard (Table 4).

**Table 4:** Recommendations regarding on‐demand treatment with clotting factors in patients with Hemophilia A or B without inhibitors, issued by clinical practice guidelines.

No	Author year (country)	Recommendations on bleeding without immediate life‐threatening	Recommendations on immediately life‐threatening bleeding
*CPGs from lower‐middle‐income countries*			
1	India‐IAP (2018)	Raise the plasma concentration of the factor to a level greater than or equal to 40–60 IU/dL in the availability of resources. In the absence of resources, consider 10–30 IU/dL. Does not mention the number of days.	Raise the plasma concentration of the factor to a level greater than or equal to 80–100 IU/dL in the availability of resources. In the absence of resources, consider 50–80 IU/dL. Does not mention the number of days.
2	GMHE 2018 (Egypt)	‐	‐
*CPGs from upper‐middle‐income countries*			
3	GMHC 2021 (Argentina)	30 IU/dL, for 2–3 days	70–100 IU/dL, for 15 days. Then maintain the factor to a level greater than or equal to 30 IU/dL.
4	MINSAL 2020 (Chile)	40–60 IU/dL, 3 to 5 days	80–100 IU/dL, 21 days
5	IMSS 2018 (Mexico)	40–60 IU/dL, 2 to 3 days or more according to response.	80–100 IU/dL, 14–21 days
6	IGSS 2017 (Guatemala)	‐	‐
7	EsSalud 2021 (Peru)	40–60 IU/dL, 3–5 days	80–100 IU/dL, 21 days
*CPGs from high‐income countries*			
8	NHG 2021 (Nordic countries)	‐	‐
9	NHC 2019 (Ireland)	50 IU/dL, does not specify the time	100 IU/dL, does not specify the time
10	BSH 2020 (United Kingdom)	‐	‐
*International CPGs*			
11	World Federation of Hemophilia (WFH) 2020 (Canada)	40–60 IU/dL, 3–5 days	80–100 IU/dL, 21 days

Abbreviation: CPG, clinical practice guideline.

Regarding life‐threatening bleeding, 5/11 CPGs^5^, ^15^, ^16^, ^17^, ^22^ recommended raising the plasma concentration of the factor to 80–100 IU/dL during 14–21 days according to the response, 1/11^21^ recommended raising the plasma concentration of the factor to ≥ 70–100 IU/dL for 15 days and then maintain the factor level ≥ 50 IU/dL, 1/11^23^ recommended raising the plasma concentration of the factor to ≥ 100 IU/dL but does not specify time, and 4/11^18^, ^19^, ^20^, ^24^ did not issue a recommendation on this regard (Table 4).

## 4 DISCUSSION

Among the 11 included hemophilia CPGs, the overall AGREE‐II mean scores ranged from 14% to 83%, and only three CPGs had a score ≥ 60%. We did not find other studies that have assessed the methodological quality of GPCs for hemophilia or other hematologic diseases. However, a systematic review that assessed 21 pediatric diabetes mellitus two CPGs also found a great heterogeneity across guidelines, with overall scores ranging from 22.0% to 90.3%.^25^ This was also observed in another systematic review that assessed seven CPGs for fever management in pediatrics and reported overall AGREE‐II scores that ranged from 34.6% to 85.9%.^26^ No pattern was detected according to the income stratum, regarding any of the domains of the AGREE‐II instrument used to assess the quality of the CPGs.

CPGs may be defined as “statements that include recommendations, intended to optimize patient care, that are informed by a systematic review of the evidence and an assessment of the benefits and harms of alternative care options.”^27^ That is to say, CPGs recommendations should be based on systematic reviews of the literature and benefits‐harms balances. However, we found that, of the 10 CPGs that issued a primary prophylaxis dose recommendation, only 3/10^15^, ^16^, ^17^ mentioned having carried out a search strategy, 2/10^5^, ^15^ showed a justification of the dose decision, and 0/11 performed an economic evaluation.

That means that most of the CPGs reached to a recommendation either without showing a justification regarding their benefits‐harms‐costs balance. This could be because such recommendations were taken without formal evidence assessments (so the recommendation may not reflect a true benefits‐harms balance of the intervention) or that even when some efforts to achieve evidence‐based decisions were made, those are not adequately reported in the guidelines (so the readers cannot understand how this recommendation was reached and when or how it may be applicable).

Regarding the dose of clotting factor concentrate for starting primary prophylaxis, most CPGs considered an intermediate or high dose of clotting factor concentrate, individualizing the treatment, and only 4/11 CPGs^5^, ^15^, ^22^, ^24^ considered the possibility of starting prophylaxis from a low dose (with individualization of the dose according to the severity of the disease, the behavior of bleeding, and/or the availability of the drug). Although the evidence suggests that intermediate or high doses provide superior results than low doses, the certainty of the evidence is very low, and there is no reliable information regarding the clinical benefits of giving a higher dose.^28^ Moreover, the high investments of giving a higher dose can translate into fewer resources assigned for other needs, causing inequity, especially for countries with limited income. So there is an urgent need for local cost analyses to compare these regimens.^29^

The decision‐making regarding the dose of clotting factor concentrate for primary prophylaxis in hemophilia is complex and reveals several shortcomings. On the one hand, the lack of evidence (lack of randomized controlled trials that have compared different doses of different types of factors), and on the other hand, shortcomings in the decision‐making process on the CPGs (no detailed information regarding the realization of the systematic reviews, benefits‐harms balances, and cost assessments, to make the decisions). This is disturbing, given the high cost involved, the inequity that this may mean for low‐income countries, and potential bias or conflicts of interest.

## 5 LIMITATIONS

Our study had certain limitations. First, although we searched CPGs without language restrictions, we used the terms English, so possibly some guides published entirely in another language (without translation of the title into English) could not have been collected. Second, we only reviewed the published information of each CPG and did not contact the CPGs authors for further details. We planned the study in that manner because we intended to evaluate the information available to any reader of the CPGs.

Our study had used limitations that should be considered. First, although we conducted a comprehensive search for CPGs without language restrictions, we used English search terms, which may have resulted in the exclusion of some CPGs published entirely in other languages (with titles not translated into English). Second, we relied solely on the published information of each CPG and did not contact the authors for further details, although we adopted this approach to evaluate the information available to any reader of the CPGs.

### 5.1 Conclusion

In conclusion, our findings indicate that the quality of hemophilia CPGs is not optimal, with inconsistent recommendations for prophylaxis and a lack of justification for these recommendations. To ensure that future CPGs are evidence‐based and trustworthy, there is a need to improve the transparency and rigor of the decision‐making process in the development of hemophilia CPGs. This will allow healthcare professionals to better understand and evaluate the applicability of the recommendations in their specific contexts.

## AUTHOR CONTRIBUTIONS

**Carolina J. Delgado‐Flores**: Conceptualization; data curation; formal analysis; funding acquisition; methodology; project administration; validation; visualization; writing—review & editing. **David García‐Gomero**: Data curation; formal analysis; investigation; methodology; writing—original draft; writing—review & editing. **Liseth Pinedo‐Castillo**: Formal analysis; investigation; visualization; writing—original draft; writing—review & editing. **Alvaro Taype‐Rondan**: Conceptualization; methodology; project administration; supervision; validation; visualization; writing—review & editing.

## CONFLICT OF INTEREST STATEMENT

Carolina J. Delgado‐Flores, David García‐Gomero, and Alvaro Taype‐Rondan participated in the elaboration of the clinical practice guideline for Hemophilia in EsSalud (Peru).

## TRANSPARENCY STATEMENT

The lead author Carolina J. Delgado‐Flores affirms that this manuscript is an honest, accurate, and transparent account of the study being reported; that no important aspects of the study have been omitted; and that any discrepancies from the study as planned (and, if relevant, registered) have been explained.

## Supporting information

Supporting information.

## Data Availability

Data supporting the findings of this study are available in the supplementary material to this article.

## References

[1] Blanchette VS , Key NS , Ljung LR , et al. Definitions in hemophilia: communication from the SSC of the ISTH. J Thromb Haemostasis. 2014;12(11):1935‐1939. 25059285 10.1111/jth.12672

[2] White G , Rosendaal F , Aledort L , et al. Definitions in hemophilia. Recommendation of the scientific subcommittee on factor VIII and factor IX of the scientific and standardization committee of the international society on thrombosis and haemostasis. Thromb Haemost. 2001;85(3):560. 11307831

[3] Iorio A , Stonebraker JS , Chambost H , et al. Establishing the prevalence and prevalence at birth of hemophilia in males: a meta‐analytic approach using national registries. Ann Intern Med. 2019;171(8):540‐546. 31499529 10.7326/M19-1208

[4] Makris M , Oldenburg J , Mauser‐Bunschoten EP , et al. The definition, diagnosis and management of mild hemophilia A: communication from the SSC of the ISTH. J Thromb Haemostasis. 2018;16(12):2530‐2533. 30430726 10.1111/jth.14315

[5] Srivastava A , Santagostino E , Dougall A , et al. WFH guidelines for the management of hemophilia, 3rd edition. Haemophilia. 2020;26(S6):1‐158. 32744769 10.1111/hae.14046

[6] Tricco AC , Lillie E , Zarin W , et al. PRISMA extension for scoping reviews (PRISMA‐ScR): checklist and explanation. Ann Intern Med. 2018;169(7):467‐473. 30178033 10.7326/M18-0850

[7] World Bank . Income level. World Development Indicators, The World Bank Group, 03 April 2022 of publication. Accessed April 03, 2022. https://data.worldbank.org/indicator/SI.POV.DDAY?locations=PE-XT

[8] Brouwers MC , Kho ME , Browman GP , et al. AGREE II: advancing guideline development, reporting and evaluation in health care. Can Med Assoc J. 2010;182(18):E839‐E842. 20603348 10.1503/cmaj.090449 PMC3001530

[9] Brosseau L , Rahman P , Toupin‐April K , et al. A systematic critical appraisal for non‐pharmacological management of osteoarthritis using the appraisal of guidelines research and evaluation II instrument. PLoS One. 2014;9(1):e82986. 24427268 10.1371/journal.pone.0082986 PMC3888378

[10] Poitras S , Avouac J , Rossignol M , et al. A critical appraisal of guidelines for the management of knee osteoarthritis using appraisal of guidelines research and evaluation criteria. Arthritis Res Ther. 2007;9(6):R126. 18062805 10.1186/ar2339 PMC2246248

[11] Yan J , Min J , Zhou B . Diagnosis of pheochromocytoma: a clinical practice guideline appraisal using AGREE II instrument. J Eval Clin Pract. 2013;19(4):626‐632. 22809219 10.1111/j.1365-2753.2012.01873.x

[12] Fuentes Padilla P , Martínez G , Vernooij RWM , Cosp XB , Alonso‐Coello P . Nutrition in critically ill adults: a systematic quality assessment of clinical practice guidelines. Clin Nutr. 2016;35(6):1219‐1225. 27068586 10.1016/j.clnu.2016.03.005

[13] Hurdowar A , Graham ID , Bayley M , Harrison M , Wood‐Dauphinee S , Bhogal S . Quality of stroke rehabilitation clinical practice guidelines. J Eval Clin Pract. 2007;13(4):657‐664. 17683311 10.1111/j.1365-2753.2007.00708.x

[14] Montesinos‐Guevara C , Andrade Miranda A , Bedoya‐Hurtado E , et al. Evaluación de la calidad de guías de práctica clínica para el tratamiento de psoriasis mediante la herramienta AGREE II. Actas Dermosifiliogr. 2022; 113(3):222‐235. 35526917 10.1016/j.ad.2021.09.004

[15] Instituto de Evaluación de Tecnologías en Salud e Investigación Guía de Práctica Clínica para Diagnóstico y Tratamiento de Hemofilia: Guía en Versión Extensa. EsSalud; 2021.

[16] Ministerio de Salud . Resumen ejecutivo guía de práctica clínica hemofilia. MINSAL; 2020.

[17] Diagnóstico y Tratamiento de Hemofilia Hereditaria en < de 16 Años . Guía de Evidencias y Recomendaciones: Guía de Práctica Clínica. México, Instituto Mexicano del Seguro Social; 2018.

[18] Instituto Guatemalteco de Seguridad Social (IGSS) . GPC‐BE 99 “manejo de hemofilia en el paciente pediátrico”. Edición. 2017; págs. 74, IGSS, Guatemala. Accessed April 03, 2022. https://fdocuments.ec/document/guia-de-prctica-clnica-2019-07-15-prctica-clnica-es-creado-con-el-propsito.html?page=3

[19] Rayment R , Chalmers E , Forsyth K , et al. Guidelines on the use of prophylactic factor replacement for children and adults with haemophilia A and B. Br J Haematol. 2020;190(5):684‐695. 32390158 10.1111/bjh.16704

[20] Nordic Hemophilia Guidelines . Nordic Hemophilia Council. Paises Nórdicos, 2020. Accessed April 03, 2022. https://www.nordhemophilia.org/library/Files/PDF-skjol/Nordic%20Hemophilia%20Guidelines_May_2020.pdf

[21] Fundación de Hemofilia de Argentina . Guía para el manejo de la Hemofilia congénita. Buenos Aires; 2021.

[22] Sachdeva A , Gunasekaran V , Ramya HN , et al. Consensus statement of the Indian academy of pediatrics in diagnosis and management of hemophilia. Indian Pediatr. 2018;55(7):582‐590. 30129541

[23] National Hemophilia Council . Adults with Haemophilia and Related Bleeding Disorders Acute Treatment Guidelines. Ireland, 2019. Accessed April 03, 2022. http://www.nationalhaemophiliacouncil.ie/

[24] Mokhtar G , El‐Beshlawy A , El Alfy M , et al. Guidelines for the management of haemophilia in Egypt. J Haemophilia Pract. 2018;5(1):83‐92.

[25] Bhatt M , Nahari A , Wang PW , et al. The quality of clinical practice guidelines for management of pediatric type 2 diabetes mellitus: a systematic review using the AGREE II instrument. Syst Rev. 2018;7(1):193. 30442196 10.1186/s13643-018-0843-1 PMC6238336

[26] Chiappini E , Bortone B , Galli L , Martino M . Guidelines for the symptomatic management of fever in children: systematic review of the literature and quality appraisal with AGREE II. BMJ Open. 2017;7(7):e015404. 10.1136/bmjopen-2016-015404 PMC5642818 28760789

[27] Consensus report, Institute of Medicine . Clinical practice guidelines we can trust. 2011. Accessed April 03, 2022. https://www.iom.edu/Reports/2011/Clinical-Practice-Guidelines-We-Can-Trust.aspx

[28] Delgado‐Flores CJ , García‐Gomero D , Salvador‐Salvador S , Montes‐Alvis J , Herrera‐Cunti C , Taype‐Rondan A. Effects of replacement therapies with clotting factors in patients with hemophilia: a systematic review and meta‐analysis. PLoS One. 2022;17(1):e0262273. 35030189 10.1371/journal.pone.0262273 PMC8759703

[29] Mannucci PM , Mancuso ME , Santagostino E . How we choose factor VIII to treat hemophilia. Blood. 2012;119(18):4108‐4114. 22411872 10.1182/blood-2012-01-394411

