# Supplementary material for: Methodological quality and recommendations of hemophilia clinical practice guidelines: A scoping review
Source: Health Sci Rep. 2023 Jul 13;6(7):e1326. doi: 10.1002/hsr2.1326 (PMC10339284; doi:10.1002/hsr2.1326)
Supplement: Supplementary file 1 — Supporting information. [file HSR2-6-e1326-s001.docx]

# Supplementary Material

## Supplementary Material 1: Search strategy

| **Sources** | **Date** | **Term** | **Results** |
| --- | --- | --- | --- |
| Trip Database | December 13, 2021 | Haemophilia | 215 |
| Base internacional de guías GRADE | December 13, 2021 | haemophilia | 4 |
| PubMed | December 14, 2021 | (haemophilia[mesh] or hemophilia[mesh] or haemophilia[tiab] or hemophilia[tiab]) and (guideline[publication type] or guideline*[TI] or recommendation*[TI] | 59 |
| Google scholar | December 14, 2021 | Haemophilia guideline guidelines  Hemofilia guía de práctica clínica | 200 |
| Google | December 14, 2021 | Haemophilia guideline guidelines  Hemofilia guía de práctica clínica | 200 |

# 
